# Integrated In-Plane Nanofluidic Devices for Resistive-Pulse Sensing

**DOI:** 10.1146/annurev-anchem-061622-030223

**Published:** 2024-07-02

**Authors:** Tanner W. Young, Michael P. Kappler, Ethan D. Call, Quintin J. Brown, Stephen C. Jacobson

**Affiliations:** Department of Chemistry, Indiana University, Bloomington, Indiana, USA

**Keywords:** nanofluidics, resistive-pulse sensing, nanofabrication, in-plane, integrated devices, biomolecules

## Abstract

Single-particle (or digital) measurements enhance sensitivity (10- to 100-fold improvement) and uncover heterogeneity within a population (one event in 100 to 10,000). Many biological systems are significantly influenced by rare or infrequent events, and determining what species is present, in what quantity, and the role of that species is critically important to unraveling many questions. To develop these measurement systems, resistive-pulse sensing is used as a label-free, single-particle detection technique and can be combined with a range of functional elements, e.g., mixers, reactors, filters, separators, and pores. Virtually, any two-dimensional layout of the micro- and nanofluidic conduits can be envisioned, designed, and fabricated in the plane of the device. Multiple nanopores in series lead to higher-precision measurements of particle size, shape, and charge, and reactions coupled directly with the particle-size measurements improve temporal response. Moreover, other detection techniques, e.g., fluorescence, are highly compatible with the in-plane format. These integrated in-plane nanofluidic devices expand the toolbox of what is possible with single-particle measurements.

## INTRODUCTION

The introduction of Coulter counting (1, 2) or resistive-pulse sensing opened the door to accurately measuring the size, shape, charge, and concentration of micro- and nanoscale particles suspended in solution. Initial devices were used to determine the concentration of platelets (3) and red and white blood cells (2, 4) in human blood. Since that time, the push to use smaller pores for resistive-pulse measurements has led to characterizing smaller particles and molecules. Advances include the use of track-etched membranes to characterize particles and viruses with diameters of ~100 nm (5, 6) and membrane protein pores immobilized in a lipid bilayer to detect single-stranded RNA and DNA molecules (7). These advances represent a 1,000-fold improvement in the size of particles and molecules that can be interrogated with resistive-pulse sensing.

Over the past few decades, nanoscale fabrication methods have improved significantly, and solid-state nanopores are a reliable platform for direct, label-free, and real-time analysis of a range of biomolecules (8–11). Fabrication techniques to form nanopores in various substrate materials include focused electron beam (e-beam) drilling (12–14), focused ion beam (FIB) milling (15–18), nanoimprint lithography (19),pipet pulling (20–22),and controlled dielectric breakdown (23,24). The size, geometry, and surface properties of solid-state nanopores are easily tuned with these fabrication methods (8, 10, 25–27). Much of the work to date has focused on forming nanopores in a free-standing membrane and perpendicular to the membrane surface, often referred to as an out-of-plane nanopore. Considerable investment has been made to optimize fabrication of out-of-plane nanopores, which routinely have diameters of single nanometers and offer exquisite sensing properties. However, integration of these nanopores into a sensing platform with connections to other fluidic components requires precise alignment of the nanopores with those components (18, 28–31).

An alternative approach is to integrate micro- and nanofluidic channels and pores in the plane of the substrate. Fabrication of these in-plane integrated fluidic components combines microfabrication to form microfluidic channels and nanofabrication to create nanofluidic channels. A set of interconnected nanoscale conduits can be fabricated directly by FIB milling into glass substrates (32–34) or replication of nanoscale features into polymers (35). With the in-plane architecture, multiple fluidic components can easily be combined in a single device without the need to align components after fabrication. In addition, these in-plane devices control fluids precisely, consume less sample, easily couple multiple detection methods (e.g., electrical and optical), and exhibit improved mass transfer of samples (31, 35, 36).

An important feature of in-plane devices is the ability to integrate multiple functions in series or in parallel to execute multistep assays of interest. A wide range of functional elements is available and includes injectors, mixers, filters, reactors, separators, sorters, collectors, and sensors. Injectors and mixers accurately meter samples and reagents into, for example, reactors for chemical transformation of the sample. Filters, separators, sorters, and collectors manipulate and direct samples within the channel architecture, and sensors detect the presence of and characterize the biophysical properties of the sample. At the nanoscale, timing among functional elements must be precise, which seamless integration ensures.

In-plane nanofluidic components can be described as plug-and-play elements that can be used as individual modules or coupled in series with one another, reordered upstream or downstream to accomplish a specific assay, or placed in parallel to enhance throughput. The possible combinations of functional elements are seemingly endless and can be considered a toolbox for measurements at the nanoscale. In this review, we discuss the principles of resistive-pulse sensing, fabrication of nanochannels and pores, examples of biophysical measurements, and integration of functional elements. We also suggest future directions and applications for these integrated devices.

## RESISTIVE-PULSE SENSING

Resistive-pulse sensing detects individual particles as they translocate through an electrically biased constriction (or pore) (1, 2). As the particle passes through the pore, a volume of electrolyte proportional to the particle volume is displaced, which leads to a change in resistance within the pore and a resulting change in the current. The measured change in current for this transient event is a current pulse with a pulse amplitude (Δi), which is proportional to the particle volume, and a pulse width or dwell time $\left(t_{d}\right)$, which is proportional to the time spent in the pore, particle shape, and electrophoretic mobility. Particles with larger volumes displace a larger volume of electrolyte from the pore and have a larger pulse amplitude. Particles with a higher surface charge density spend less time in the pore and have a shorter dwell time. Moreover, the frequency of the current pulses is proportional to the particle concentration within the sample.

The measured current pulse amplitude in resistive-pulse sensing depends, to first order, on the volume of the particle relative to the volume of the pore. The change in resistance within a pore (ΔR) is calculated from the volume of electrolyte displaced by a nonconducting particle relative to the pore volume (37):

1. $$\Delta R=\frac{4\rho d^{3}}{\pi D^{4}},$$

where ρ,d, and D are the solution resistivity, particle diameter, and pore diameter, respectively. This relationship is valid when the particle diameter is much smaller than the pore diameter. The signal-to-noise ratio of the measurement improves as the pore diameter approaches the particle diameter. However, as the pore diameter approaches the particle diameter, the electric field lines begin to bulge around the particle, leading to an increased change in resistance relative to what is expected with Maxwell’s approximation (Equation 1). To overcome this limitation, DeBlois et al. (37) account for the electric field line distortions:

2. $$\Delta R=\frac{4\rho d^{3}}{\pi D^{4}}\frac{1}{\left(1\;-\;0.8{\left(\frac{d}{D}\right)}^{3}\right)}.$$

This modification to the equation calculates ΔR to within 1% for a particle diameter that is 10% to 80% of the pore diameter.

### Fabrication of In-Plane Devices

**Figure 1:**
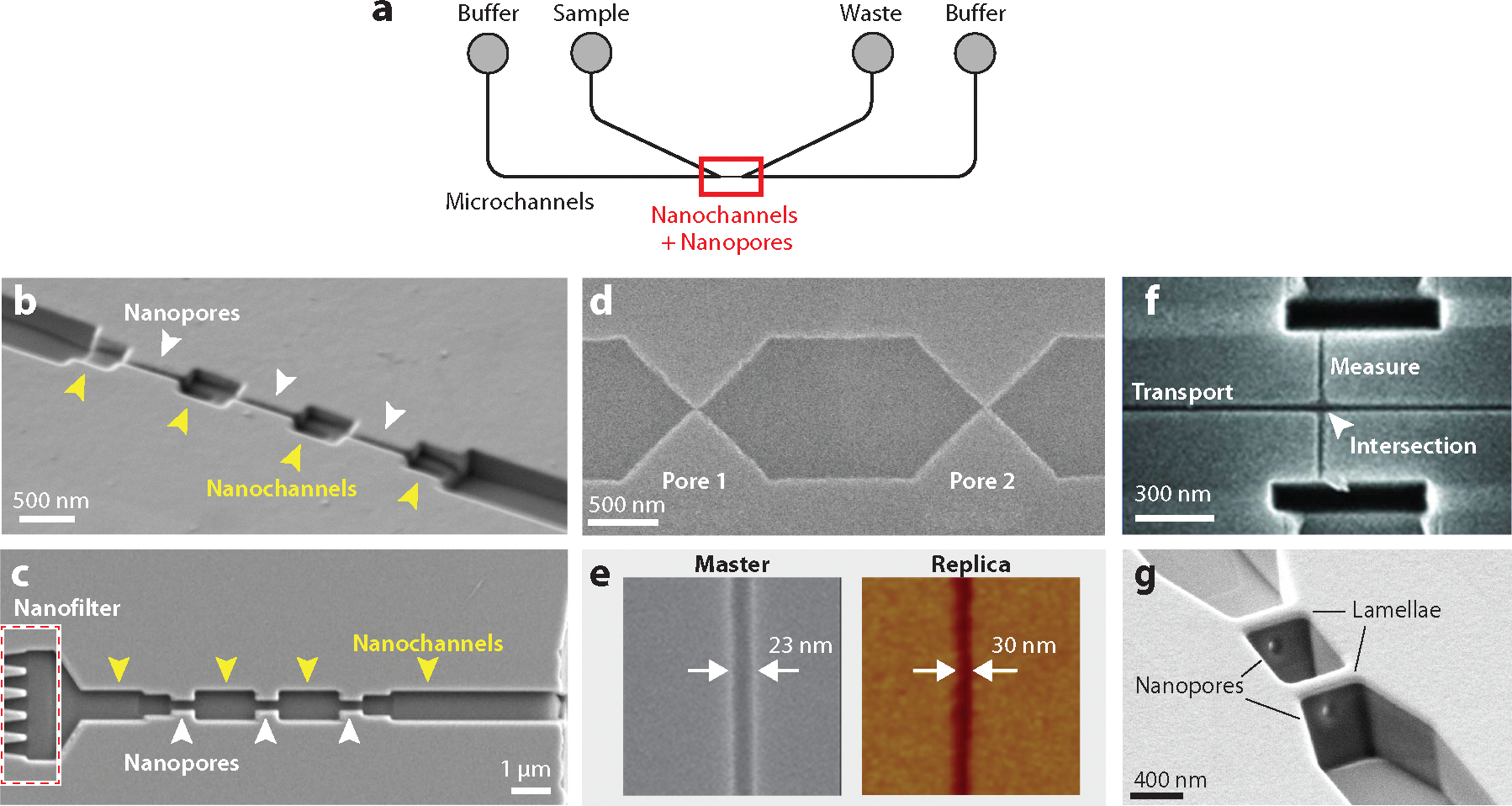
Nanofluidic nanochannels and nanopores. (*a*) Schematic of microchannels designed with a small gap (10–100 μm) between them in which nanochannels and nanopores are fabricated by the preferred nanofabrication technique (*red box*). (*b*) Scanning electron microscope (SEM) image of three nanopores in series connected by two pore-to-pore nanochannels and fabricated in a glass substrate by focused ion beam (FIB) milling to measure hepatitis B virus capsids (Figure 2a,b). (*c*) SEM image of three pores in series with two pore-to-pore nanochannels fabricated in a glass substrate by FIB milling to measure extracellular vesicles (Figure 2c,d). This design included an upstream nanoscale filter to prevent cellular debris and aggregates from entering the detection region. Panel *c* adapted with permission from Reference 43; copyright 2023 American Chemical Society. (*d*) SEM image of two nanopores in series fabricated in a silicon substrate by electron beam (e-beam) lithography and reactive ion etching. Panel *d* adapted with permission from Reference 44; copyright 2011 American Chemical Society. (*e*) SEM image of a silicon master and atomic force microscope image of a polymer replica of a nanochannel fabricated by the master-mold-replica process. Panel *e* adapted with permission from Reference 46; copyright 2011 American Vacuum Society. (*f*) SEM image of a nanofluidic cross intersection fabricated in a glass substrate by FIB milling for trans-conductance measurements of DNA electrokinetically migrating through the transport channel and measured at the intersection through the measure channel. Panel *f* adapted with permission from Reference 49; copyright 2012 American Chemical Society. (*g*) SEM image of two nanopores with circular cross sections milled with an FIB instrument into tilted lamellae formed in a nanochannel. Panel *g* adapted with permission from Reference 50; copyright 2022 American Chemical Society.

To achieve high-quality resistive-pulse data, nanopores must be fabricated with dimensions comparable to the particle of interest. Over the past two decades, fabrication techniques, including FIB milling, e-beam lithography, and nanoimprint lithography, have continued to improve and provide robust fabrication methods for both in-plane and out-of-plane nanofluidic devices. A common strategy for designing and fabricating in-plane devices is to create a set of two or more microchannels with conventional photolithography and etching techniques, but leave small gaps, e.g., 10–100 μm, between adjacent microchannels into which nanoscale channels and pores are fabricated with the preferred technique (Figure 1a). The pores are the elements that define the critical dimensions for detection and typically have dimensions from a few nanometers to a few micrometers (32, 38). The in-plane architecture allows for nearly seamless integration of multiple pores in series and other functional elements to measure single particles.

### Nanofabrication Techniques

As noted above, several nanofabrication techniques are available to fabricate nanochannels and nanopores on in-plane devices. The most versatile of the techniques is FIB milling, which provides excellent control over three-dimensional (3D) topography of the channel and pore dimensions. Through computer-aided design (CAD) software, nanoscale features are designed, and the ion beam doses to mill those features are set. Gradients of the ion beam dose can mill continuous features, e.g., 3D funnels, straightforwardly (39). Moreover, nanochannels thinner than the beam width have even been milled into substrates (32) by increasing the thickness of a protective metal film. With FIB milling, implantation of metal ions, e.g., gallium (Ga), into the substrate can alter the surface characteristics (40). After milling, Ga ions can be removed by soaking the substrate in water (41), which is time consuming (1–3 days), but the milled nanochannels and nanopores retain their dimensions and have electroosmotic flow similar to that of the original glass surface. Ga ions can also be removed more rapidly (~1 h) with an HCl-based etchant (42), but the channels and pores are etched, and their dimensions increase up to the penetration depth of the Ga ions (up to ~30 nm).

Figure 1b,c displays scanning electron microscope (SEM) images of a series of three nanopores milled into a glass substrate for resistive-pulse sensing (43). With the in-plane architecture, designing and milling multiple nanopores in series are straightforward, and coupling of multiple nanopores in series improves the precision of the resistive-pulse measurements (discussed below in the section titled Resistive-Pulse Measurements). A second approach to fabricate nanochannels and nanopores is to combine e-beam lithography with reactive ion etching (RIE). E-beam lithography is used to pattern features serially, which are then etched in parallel with RIE. Figure 1d shows an SEM image of a device with two pores fabricated with e-beam lithography and RIE (44). The nanopores are 50 nm wide and 50 nm deep and were used to sense hepatitis B virus (HBV) capsids. Although e-beam lithography can generate patterns more rapidly than FIB milling, features are etched to a uniform depth during the RIE step. Consequently, 3D structures require precise alignment of multiple lithography and etching steps, which are more difficult to execute.

Both FIB milling and e-beam lithography produce features at nanometer-length scales with excellent precision, but both methods are serial processes. To improve device throughput, nanofluidic devices can be cast or imprinted into polymer substrates through a master-replica process (45) or a master-mold-replica process (46). With the master-replica process, a negative master is produced by, for example, e-beam lithography of a negative-tone resist. This master can be further shaped by e-beam induced etching. The replica is then cast into a polymer, e.g., high-modulus poly(dimethylsiloxane). Devices with nanopores and funnels with 3D geometries have been fabricated with this method.

With the master-mold-replica process, the positive master is fabricated by e-beam lithography, FIB milling, or both. A negative mold is formed from the master, and replicas are then cast or imprinted from the mold. Figure 1e shows a nanochannel fabricated by this double replication process (46). Nanochannels and nanopores with dimensions as small as 10 nm can be fabricated with this two-step process (47). For both replication processes, the most time-consuming step is creation of the initial master from which multiple molds and subsequent replicas can be cast or imprinted. Typically, the Young’s modulus of a polymer is substantially lower than a glass or silicon substrate; consequently, the polymer must have sufficient cross-linking to prevent the nanoscale features from collapsing. Also, the options to modify the surface of a nanochannel formed in a polymer are much fewer than those available to modify glass surfaces, which can be readily modified with silane chemistries.

### Nanopore Architectures

Because the in-plane architecture allows any two-dimensional design to be patterned onto a substrate, more complex channel and pore designs can be formed in-plane compared to out-of-plane pores, which are essentially limited to two pores in series (48). The most common strategy for detection with in-plane nanopores is fabricating multiple pores in series (Figure 1b–d). As a single particle translocates through multiple pores, the particle is interrogated multiple times. When the signals are averaged, the precision of the measurement of their physical properties improves (discussed below in the section titled Resistive-Pulse Measurements), relative to a single measurement. The depths and widths of the nanochannels and nanopores are easily tunable, allowing for passage of a range of particle sizes. Another strategy for particle sensing is to make the current measurement laterally, rather than axially. Menard et al. (49) fabricated a nanoscale cross intersection (Figure 1f), in which a DNA sample migrated through one channel, and the current was measured through the second perpendicular channel at the intersection. This strategy decouples the axial transport of the sample from the lateral measurement of the current.

In the examples above, the in-plane nanopores are milled top-down or perpendicularly to the substrate surface; consequently, the pores tend to have a rectangular or U-shaped cross section (31, 34, 36). For a spherical particle, the corners of a rectangular pore are inaccessible, which reduces the signal-to-noise ratio of the measurement, whereas the same particle can access the entire cross section of a circular pore, which enhances the signal-to-noise ratio. To generate nanopores with circular cross sections in an in-plane device, lamellae are formed within an in-plane nanochannel, and nanopores are drilled through the lamellae (50) (Figure 1g). The geometries of the lamellae and nanopores are impacted by the incidence angle and dose of the ion beam. Pore diameter increases with ion beam dose but decreases with lamella thickness when milled at constant dose. Nanopores with diameters of 30–100 nm were formed in the lamellae. Dimensions of these pores can be further reduced by annealing the surface adjacent to the pore with an e-beam (51), which provides tunability and greater control of the nanopore dimensions.

## RESISTIVE-PULSE MEASUREMENTS

**Figure 2:**
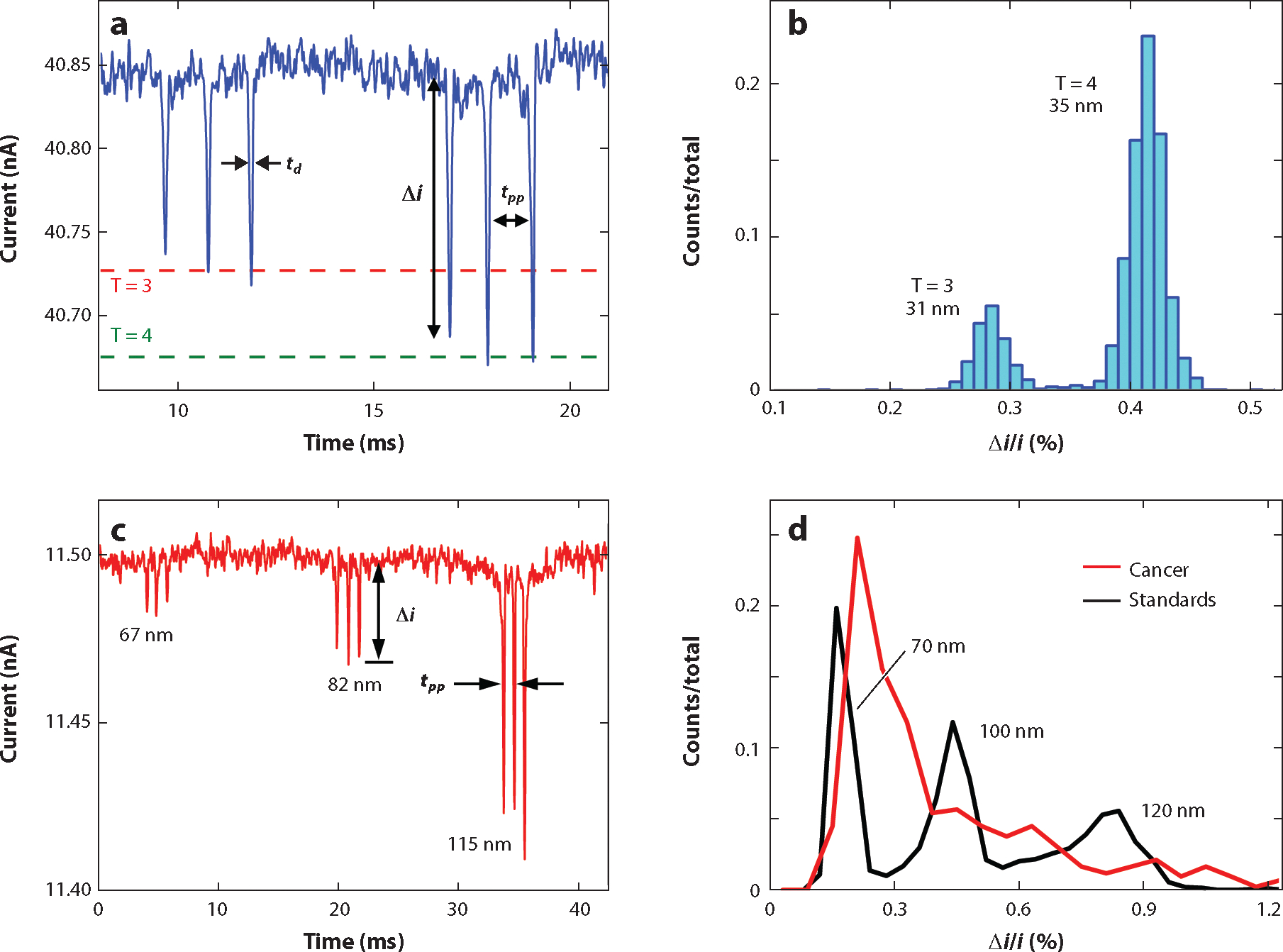
Current traces with resistive-pulse events and histograms of size distributions from resistive-pulse measurements. (*a*) Two three-pulse events for T = 3 and T = 4 hepatitis B virus (HBV) capsids translocating through the three-pore device in Figure 1b. Extracted information includes pulse amplitude (Δi), dwell time $\left(t_{d}\right)$, pore-to-pore time $\left(t_{pp}\right)$, and baseline current (i; not labeled). The triangulation number (T) describes the icosahedral structure of the capsid. (*b*) Histogram of the relative pulse amplitudes (Δi/i) for T = 3 and T = 4 HBV capsids from the data in panel *a*. Diameters of the T = 3 and T = 4 HBV capsids are 31 and 35 nm, respectively. (*c*) Three three-pulse events for extracellular vesicles (EVs) derived from a cancer cell line translocating through the three-pore device in Figure 1c. The EV diameters are 67, 82, and 115 nm. (*d*) Histograms of the relative pulse amplitudes for the EVs from the cancer cell line from the data in panel *c* and standards of polystyrene particles with diameters of 70, 100, and 120 nm. Panels *c* and *d* adapted with permission from Reference 43; copyright 2023 American Chemical Society.

Resistive-pulse measurements are conducted on devices with one or more pores in series. Figure 2a shows a current trace from a three-pore device (Figure 1b). The current trace has three pulses associated with a T = 3 capsid (31 nm in diameter) and three pulses associated with a T = 4 capsid (35 nm in diameter) traversing the three pores in series. From these measurements, the pulse amplitude (Δi), baseline current (i), dwell time $\left(t_{d}\right)$, and pore-to-pore time $\left(t_{pp}\right)$ are extracted. As noted above, the pulse amplitude corresponds to the particle volume, and the dwell time corresponds to the shape and electrophoretic mobility of the particle. With multiple pores in series, the pore-to-pore time returns the electrophoretic mobility of the particle and is used to ensure the pulse events are correlated with each other. From the pulse amplitudes, a histogram of the measured particle volumes is generated (Figure 2b). The relative pulse amplitude (Δi/i) is used to normalize measurements across devices.

Increasing the number of pores in series improves the precision of the resistive-pulse measurements. Kondylis et al. (52) illustrated the benefits of signal averaging with resistive-pulse sensing devices by fabricating and evaluating devices with two, four, and eight pores in series. A 2.7-fold decrease in the relative standard deviation (σ/mean) of the pulse amplitude distributions was observed when the amplitudes of eight pulses were averaged compared to the amplitudes of single pulses. The pore-to-pore time distributions showed a similar 3.2-fold decrease in their relative standard deviations when seven measurements from eight-pore devices were averaged in contrast to single measurements from two-pore devices. The uncertainty in the measurements decreases with signal averaging, and the resolution (mean/σ) improves by a factor equal to the square root of the number of measurements.

In addition to measuring particle size, electrophoretic mobility and, subsequently, zeta (ζ-) potential for individual particles can be extracted from the dwell time, pore-to-pore time, or both. In one example, the size and ζ-potential of extracellular vesicles (EVs), which are cell-derived, membrane-bound, nanoscale particles linked to cell–cell communication and propagation of diseases, were characterized (43) (Figure 2c,d). The in-plane devices were fabricated with three nanopores in series to determine particle diameter and two pore-to-pore regions to measure the ζ-potential. The device also included an in-line nanoscale filter to prevent cellular debris and aggregates from entering the nanopore detection region (Figure 1c). To minimize electroosmotic flow and obtain an accurate measurement of the ζ-potential, the nanochannels and nanopores were coated with a short-chain poly(ethylene glycol) silane (PEG-silane) (53). EVs from bovine milk and human breast cancer cells had similar particle size distributions (60–160 nm in diameter), but their ζ-potentials differed by twofold. The pore-to-pore time, rather than the dwell time, leads to a more accurate estimation of the ζ-potential because interactions of the particles with the nanochannel wall are less likely than with the nanopore wall. The dwell time, however, provides information about particle length if the baseline width of the current pulse is used to determine when the front of the particle enters the pore and the rear of the particle exits the pore (54).

Although increasing the number of pores in series improves measurement precision, the electrical resistance of the detection region increases, which leads to a worse limit of detection. An alternative approach to achieve multiple measurements of single particles is to drive individual particles forward and backward through multiple pores in series (i.e., particle ping-pong) (55, 56).

Cycling particles forward and backward through single nanopores is used to control and analyze DNA molecules (57–59) and nanoparticles (60, 61). The advantage to cycling particles through multiple pores is that each particle generates a unique multipulse signature, which is more easily tracked by the data analysis software. Theoretically, an unlimited number of measurements for each particle can be made, but particles are sometimes lost during the cycling if they diffuse too far from the pore before returning. Similar to having more pores in series, cycling of particles forward and backward through one or more pores provides the benefit of improved measurement precision.

**Figure 3:**
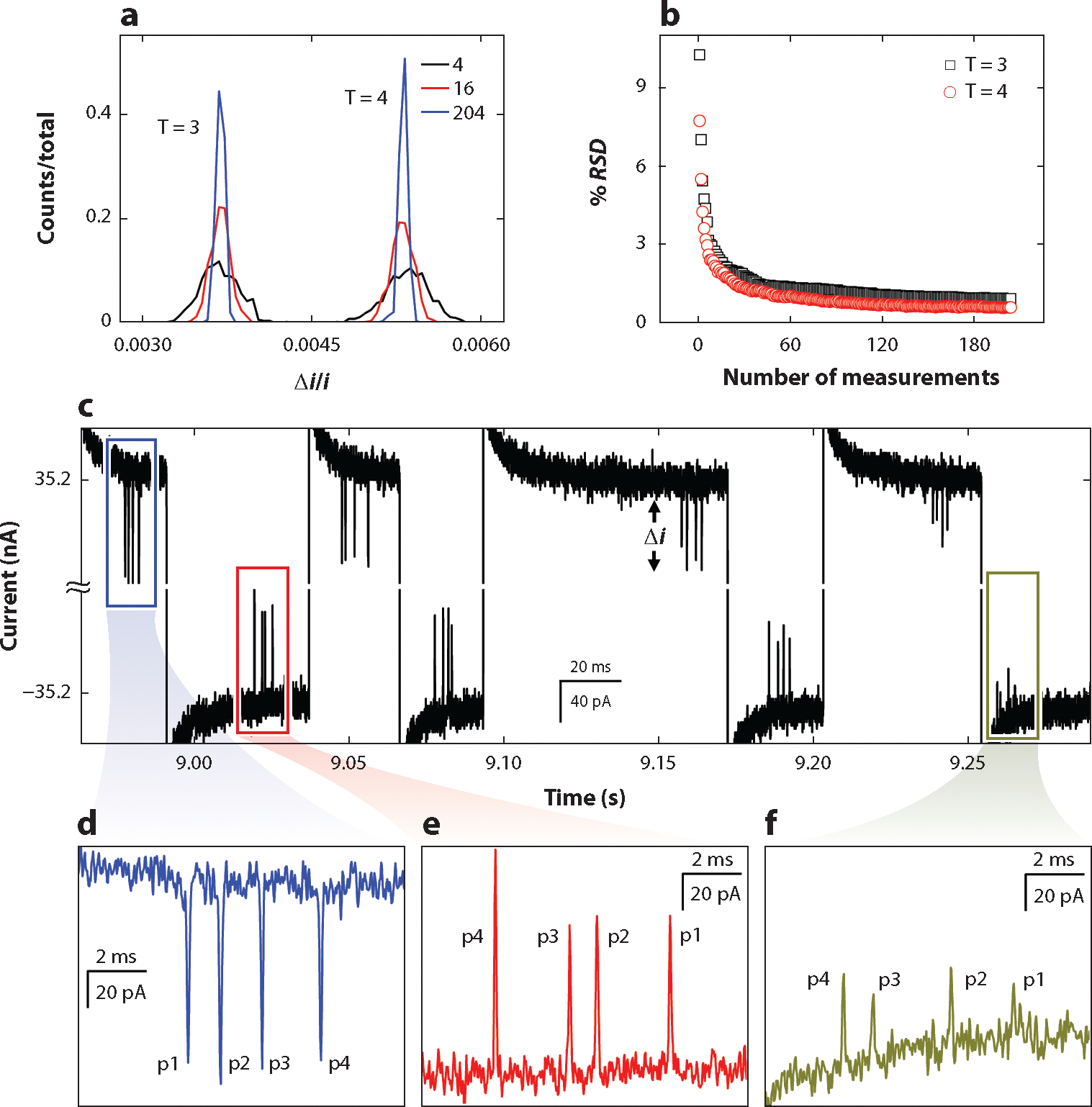
Multicycle resistive-pulse sensing (i.e., single-particle ping-pong). (*a*) Histograms of the relative pulse amplitude for resistive-pulse measurements of T = 3 and T = 4 hepatitis B virus (HBV) capsids cycled forward and backward through a four-pore device. The distributions are from 4, 16, and 204 measurements of each particle, which correspond to 0.5, 2, and 25.5 cycles, respectively. (*b*) Variation of relative standard deviation (RSD; σ/mean) with number of measurements for multicycle resistive-pulse sensing. For independent measurements, the RSD improves as one over the square root of the number of measurements. Panels *a*, *b* adapted with permission from Reference 55; copyright 2018 American Chemical Society. (*c*) The last four cycles of an HBV capsid during disassembly in the presence of guanidine hydrochloride (GuHCl). The particle progressively becomes smaller in volume from panel *d* to panel *e* to panel *f* as the capsid undergoes disassembly while passing forward and backward through pores (p) 1, 2, 3, and 4. Panels *c–f* adapted with permission from Reference 56; copyright 2022 American Chemical Society.

Figure3a shows how the histograms of the relative pulse amplitude for 4, 16, and 204 resistive-pulse measurements narrow as the number of measurements increases. The precision improves as the inverse of the square root of the number of measurements. The advantage of the ping-pong experiment is that 100 measurements of a single particle are easily obtained, which leads to greater improvement in the measurement precision. After 85 measurements, the relative standard deviation improved to less than 1% (Figure 3b). Cycling a particle multiple times through the same pores certainly provides higher precision but requires additional time to collect data. If the amount of time needed for data acquisition is a constraint, passing particles through a series of nanopores once offers improved precision along with shorter acquisition times. Higher particle throughput is gained by placing the nanopores in parallel (62). However, the relative change in resistance of a particle passing through pores in parallel is smaller, which leads to a lower detection sensitivity and a worse limit of detection.

Another method to increase the dynamic range of in-plane resistive-pulse sensing devices is node pore sensing (38), which employs a long narrow pore along which several nodes are inserted. Each node is an expanded circular section of the pore with a larger cross-sectional area, which creates a region of low resistance. The baseline current increases as a particle passes from the narrow region to the node region of the pore and decreases as the particle moves from the node region back to the narrow region of the pore. Each particle that traverses the nanochannel and nodes produces a unique signal, which is then analyzed with a fast Fourier transform to extract information about the particle. This technique offers a wide dynamic range where colloidal samples ranging from 100 nm to 5 μm in diameter were sized (38). With microscale channels, this technique can determine the mechanical properties of cells (63) and antigen–antibody interactions with cells (64).

Nanofluidic devices for lateral conductance measurements (Figure 1f) were used to measure the translocation of DNA through a long nanochannel while measuring the change in ionic conductance through a shorter channel placed orthogonally to the transport channel (49). As DNA passed the intersection, the transverse conductance was altered, which results in a current pulse. The lateral conductance exhibited a current enhancement of 5–25%, relative to the ionic current measured along the axis of DNA transport. Translocation events were confirmed with simultaneous optical and electrical detection of DNA translocation through the nanoscale cross intersection.

### Monitoring Reactions with Resistive-Pulse Sensing

Resistive-pulse sensing on in-plane nanofluidic devices with multiple pores in series has been applied to a variety of samples to characterize single particles. Since the first demonstration of sensing on in-plane multipore devices (44), work characterizing HBV has evolved from measuring fully formed capsids to monitoring assembly reactions with and without assembly effectors present and tracking disassembly of capsids exposed to chaotropes.

With respect to assembly, resistive-pulse sensing was used to characterize the formation of T = 3 and T = 4 HBV capsids from core protein dimers in real time and at biologically relevant concentrations (34, 52). Assembly reactions across a range of dimer concentrations revealed three distinct patterns. Below the pseudocritical dimer concentration of 0.5 μM, where the dimer and capsid concentrations are approximately equal, the ratio of T = 3 to T = 4 capsids increased with decreasing dimer concentration. Above the pseudocritical dimer concentration, T = 4 particles assembled rapidly, but kinetically trapped and incomplete products were also present, which slowly annealed into T = 4 capsids. At all dimer concentrations tested, T = 3 and T = 4 capsids formed at different rates, which suggests that their assembly pathways are distinct.

Core protein allosteric modulators (CpAMs) often accelerate the assembly of HBV capsids and can generate aberrant capsid morphologies, which indicates their potential as antivirals against chronic infection. Resistive-pulse sensing easily monitors the assembly products formed in the presence of CpAMs to determine their mechanism of action (54, 65). Characterization of misdirected assembly is challenging because the particles generated tend to be larger in size and nonuniform in shape. Competition between normal and CpAM-induced aberrant assembly is a function of the strength of the association energy between individual core proteins, which is proportional to ionic strength. At high ionic strength (e.g., 1 M NaCl), assembly reactions produced morphologically normal HBV capsids, even in the presence of HAP-TAMRA, which is in the family of heteroaryldihydropyrimidines (HAPs) (54). At low ionic strength (e.g., 80 mM NaCl), HAP-TAMRA led to increased particle sizes with aberrant morphologies. The smallest particles were T = 4 icosahedra, whereas the larger particles were defective spheres and ellipsoids with areas of T = 4 structure interspersed among flat regions.

To study the disassembly of HBV capsids, T = 3 and T = 4 HBV capsids were mixed with guanidine hydrochloride (GuHCl; 1.0–3.0 M), and the reaction was monitored over time by randomly selecting particles and measuring their size with resistive-pulse sensing (56). To increase the observation time and likelihood of observing a disassembly event, particles were cycled forward and backward for up to 60 s on a device with four pores in series. Figure 3c–f depicts the last four cycles of a T = 4 HBV capsid while disassembling. The pulse amplitudes of the capsid decrease from panel *d* to panel *e* to panel *f*, after which the particle is too small to be measured. At low GuHCl concentrations (e.g., 1.0 M GuHCl), capsids showed little disassembly, whereas at higher GuHCl concentrations (≥1.5 M), capsids exhibited disassembly, sometimes in a stepwise fashion. Interestingly, the disassembly process accelerated in all cases, where capsids fell apart catastrophically within 100 ms of reaching a threshold in their stability.

## INTEGRATED DEVICES

### Integration of Mixers and Reactors with Nanofluidic Devices

With in-plane devices that only incorporate resistive-pulse sensing, the products of certain reactions (e.g., assembly and disassembly of HBV capsids) have been detected and categorized, as mentioned above. In these previous experiments, reagents were mixed off-device and, subsequently, loaded onto the device. Mixing of electrolyte and core protein dimer off-device and then loading onto the device takes time, 1–2 min, and early time points in the reaction are lost. However, by integrating the reactions directly on-device with a clearly defined starting time (mixing of reagents) and stopping time (detection of products), early time points and associated short-lived intermediates in the reaction are easily monitored.

**Figure 4:**
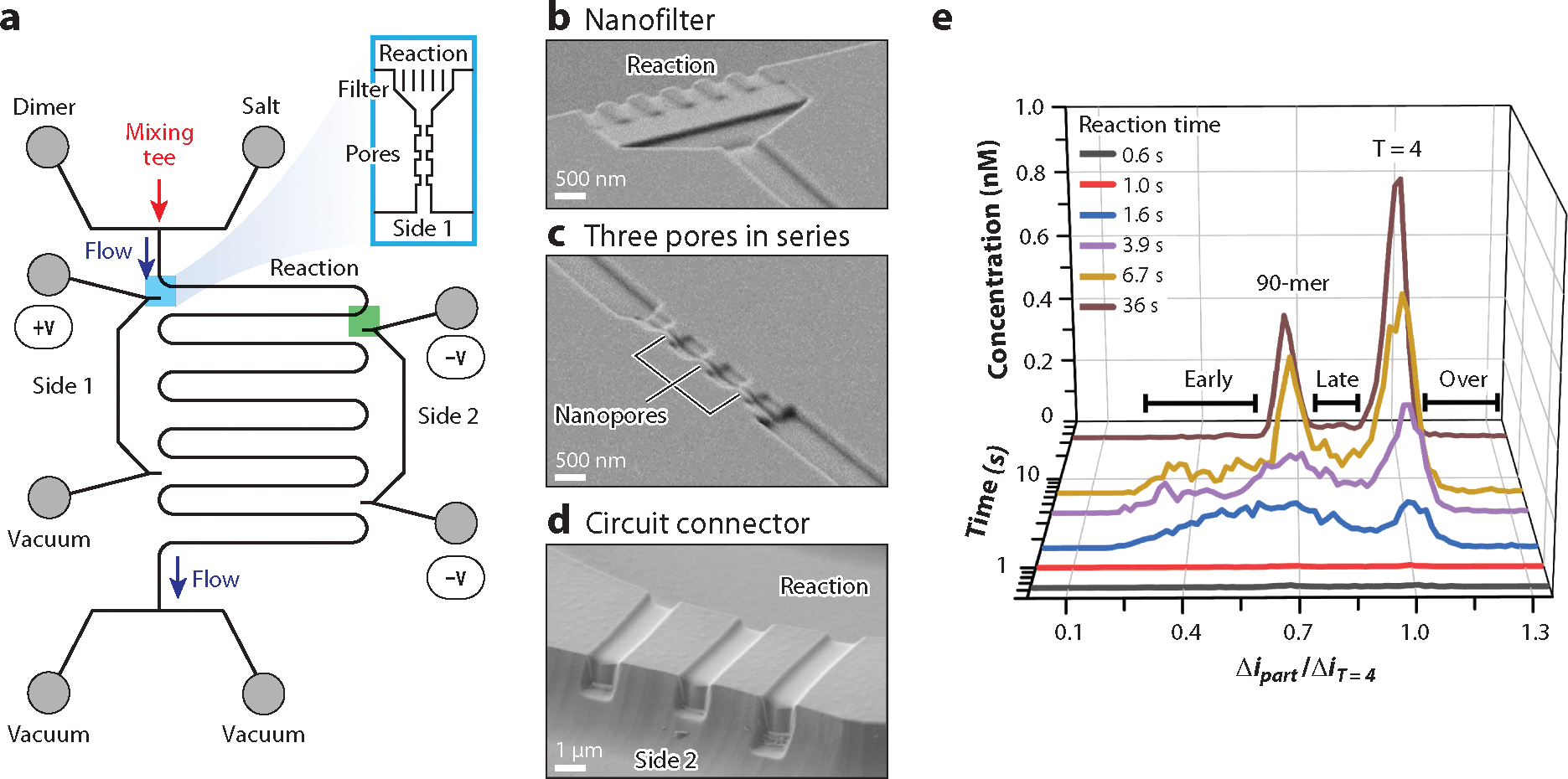
Resistive-pulse sensing integrated with on-device reactions. (*a*) Schematic of a microfluidic device with a microfluidic mixing tee, serpentine reaction channel, and resistive-pulse detection to conduct hepatitis B virus (HBV) assembly reactions. Inset shows the detection region consisting of the nanoscale filter and three nanopores in series and connecting the reaction and side 1 microchannels (*blue rectangle*). Scanning electron microscope images of (*b*) a nanoscale filter to prevent aggregates from entering the pores, (*c*) three pores in series for resistive-pulse sensing, and (*d*) circuit connector to complete the sensing circuit between +V and −V, which is three parallel nanochannels to connect the reaction and side 2 microchannels (*green square* in panel *a*). The nanopores, filter, and connector can be positioned at any of eight locations between the reaction and side 1 microchannels or the reaction and side 2 microchannels in panel *a*. (*e*) Variation of the concentration of products with normalized pulse amplitude ($\Delta i_{part}/\Delta i_{T=4}$) and reaction time (0.6 to 36 s) for assembly of HBV in 0.8 M NaCl. Particle sizes are grouped as early intermediates, 90-mers including T = 3 capsids, late intermediates, T = 4 capsids, and oversized particles. The normalized pulse amplitude is the pulse amplitude of the particle ($\Delta i_{part}$) relative to the pulse amplitude of the T = 4 capsid $\left(\Delta i_{T=4}\right)$.

**Figure 5:**
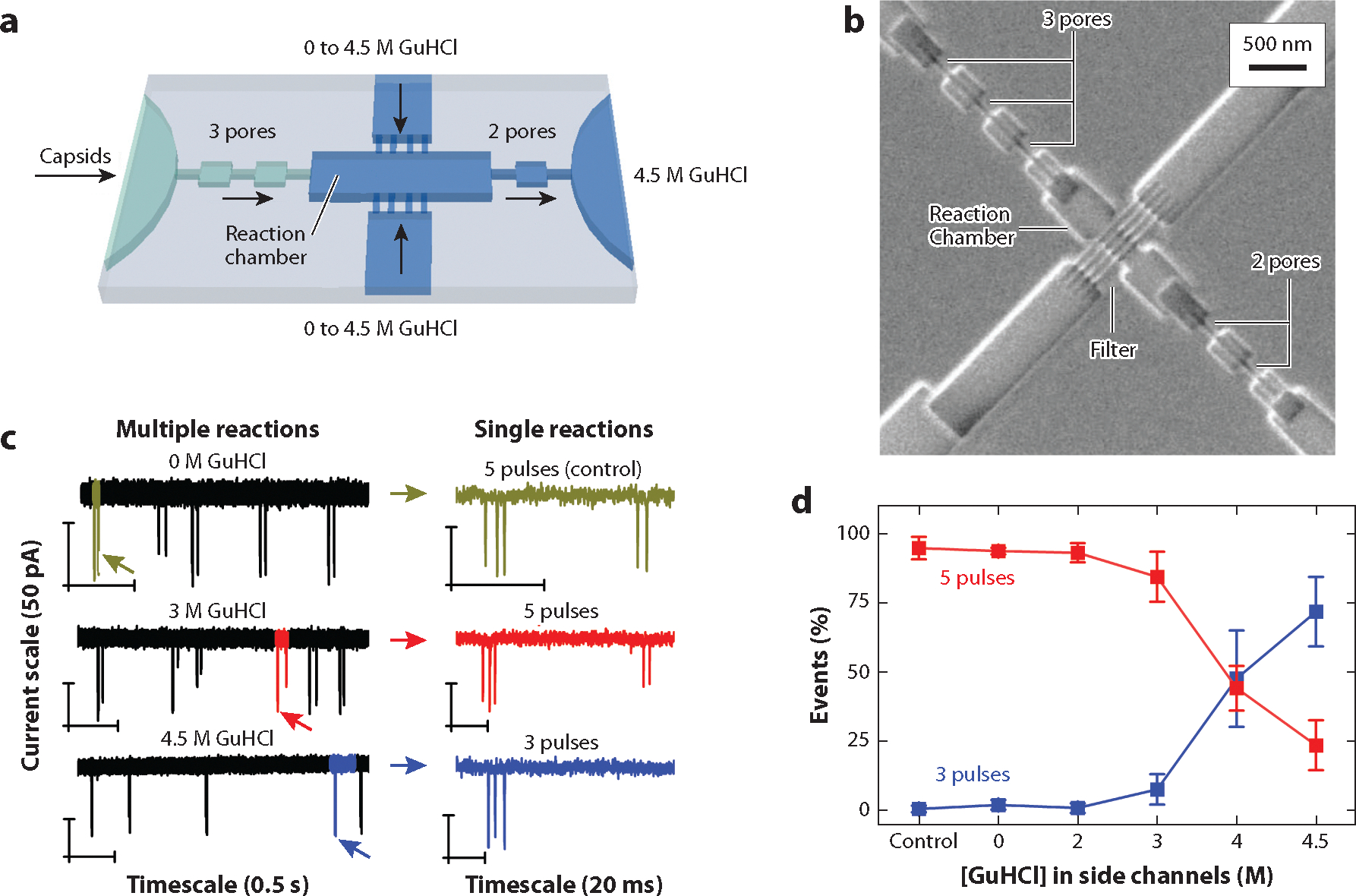
Sense-react-sense device for tracking disassembly of individual hepatitis B virus (HBV) capsids. (*a*) Schematic of the nanofluidic system used to disassemble HBV capsids where capsids are sensed prior to reaction (three-pore region), reacted with 0–4.5 M guanidine hydrochloride (GuHCl) in the reaction chamber, and sensed after the reaction (two-pore region). (*b*) Scanning electron microscope image of the sense-react-sense device with a three-pore region, reaction chamber, two filters, two side nanochannels, and two-pore region. (*c*) Current traces showing pulses of multiple and single reactions of HBV capsids mixed with 0 M, 3 M, and 4.5 M GuHCl in the reaction chamber. (*d*) Variation of the percentage of events with concentration of GuHCl for capsids that disassemble (3 pulses) and partially or do not disassemble (5 pulses).

On the microscale, mixing tees achieve mixing times on the millisecond timescale (66, 67). Rapid mixing times allow for reactions to start at specific times and locations on a microfluidic device, which provide more precise measurements of the products from the reaction at distinct times. Other examples of reactions performed in microfluidic devices include organic synthesis (68), nanocrystal synthesis (69), and reactions within droplets (70). To capture early time points in the HBV capsid assembly process, a microfluidic mixer and serpentine reaction channel have been coupled with resistive-pulse sensing to track the size and charge of intermediates and capsids (Figure 4). To monitor disassembly, a nanofluidic mixer and reaction chamber were combined with resistive-pulse sensing of the capsid prior to and following their reaction in a sense-react-sense configuration (Figure 5).

For assembly of HBV, dimer in 50 mM HEPES buffer was mixed in equal volumes with a salt solution (e.g., 1.6 M NaCl) at a mixing tee to initiate the reaction (Figure 4). As the dimer in 0.8 M NaCl flows down the reaction channel, HBV capsids form and are detected by resistive-pulse sensing at various locations along the serpentine channel (Figure 4a). The microfluidic device was designed so the resistive-pulse sensing region could be placed in eight separate locations. The reaction times on this device varied from subsecond to several minutes and depend on the location of the detection region and flow velocity of the reaction mixture traveling down the serpentine channel. Each detection region (see the inset of Figure 4a) comprises a nanoscale filter in front of the nanopores to prevent aggregates from entering the nanopores (Figure 4b) and a series of three nanopores to detect partially and fully formed capsids (Figure 4c). The electrical connection is closed with the addition of three connecting nanochannels (Figure 4d).

These experiments mimic a stop-flow reaction in which a fixed reaction time is monitored over time. Reactions were conducted at time points from 0.6 s to 36 s and compared to a 24-h assembly reaction, at which point HBV capsid assembly is considered to have reached a steady state. In Figure 4e, histograms show assembled particles of interest, as early intermediates, 90-dimer particles including T = 3 capsids, late intermediates, T = 4 capsids, and overgrown particles, and depict the evolution of those particles from 0.6 s to 36 s. Intermediates less than 90 dimers in size evolved at early time points (<10 s), formed complete capsids, and disappeared by 36 s. Similarly, late-stage intermediates arrived shortly thereafter and also disappeared. The fully formed T = 3 and T = 4 capsids evolved over time and slowly increased in concentration over the 24-h time period. Critical kinetic values, such as lag phase, are directly observed because concentrations of early intermediates, late intermediates, and fully formed capsids concentrations are monitored with time.

In disassembly of HBV, individual capsids are disassembled through exposure to the chaotrope GuHCl. The device design, referred to as a sense-react-sense configuration, consists of a first set of nanopores to measure the capsid size prior to the reaction, a nanoscale reactor to initiate disassembly of the capsid, two side channels to introduce the reagent, GuHCl, and then a second set of nanopores to measure the capsid size after the reaction (Figure 5a,b). HBV capsids and GuHCl are drawn through the device with an electric field and vacuum. Prior to exposure to GuHCl, the initial size of the capsid is measured with the three-pore sensing region. Immediately thereafter, the capsid enters the reaction chamber and is mixed with different concentrations of GuHCl, introduced from two side nanochannels, to initiate capsid disassembly. Nanofilters are placed along the side walls of the reaction chamber to prevent capsids from diffusing from the chamber. Finally, the two-pore sensing region detects the disassembly products. The average pulse amplitudes from the second two-pore region relative to the initial threepore detection region determine the extent of disassembly. No change in the pulse amplitudes between the two regions denotes disassembly had not occurred, whereas attenuated pulse amplitudes or no pulses in the second two-pore region indicate partial or complete disassembly, respectively.

The translocation time from the exit of the first set of pores to the entrance of the second set of pores is the reaction time and was ~60 ms for the experiments illustrated in Figure 5. The current traces in Figure 5c show results from experiments where the capsids were exposed to 0 M, 3 M, and 4.5 M GuHCl. The capsids remained intact with 0 M GuHCl, partially disassembled with 3 M GuHCl, and fully disassembled with 4.5 M GuHCl. For partially disassembled capsids, the two-pulse sequence after the reaction has smaller pulse amplitudes than the three-pulse sequence prior to the reaction (Figure 5c). In Figure 5d, the percentage of three- and five-pulse events is plotted with GuHCl concentration. As expected, the number of intact capsids (five pulses) decreased, and the number of disassembled capsids (three pulses) increased with increasing GuHCl concentration.

### Integrated Nanoscale Filters

Filters are another functional element that can be easily integrated with a resistive-pulse sensing device. When the sample of interest is complex or prone to aggregation, filters prevent large particles or aggregates from entering the nanopores and can increase the longevity of devices. The minimum dimension of the filter element, e.g., channel depth, is typically comparable in dimension to the nanopore, e.g., depth or width. A filter is often designed as a thin slit or an array of nanochannels (Figures 1c,4b, and 5b). Importantly, the filter should not add significantly to the overall resistance of the sensing region, should have sufficient surface area to accumulate oversized particles without clogging, and should allow particles of interest multiple pathways to enter the sensing region. The filter element in Figure 1c excludes cellular debris and aggregates for sensing extracellular vesicles. On the microscale, filters similarly exclude cellular debris and aggregates to make resistive-pulse measurements of cells (71). The nanoscale filter in Figure 4b prevents aggregates and oversized HBV particles formed during assembly experiments from entering the nanopores, whereas the nanoscale filters in Figure 5b ensure the HBV capsids remain in the reaction chamber.

### Coupling of Optical Techniques with Resistive-Pulse Sensing

In addition to integrating multiple functional elements in a single device, coupling multiple measurement techniques together can be used to extract more information from the system being studied. The in-plane format and substrate material, e.g., glass, of these nanofluidic devices are highly compatible with optical techniques, such as fluorescence imaging of particles within the nanofluidic devices and trapping of particles to conduct multiple measurements. Microfluidic devices couple well with optical techniques owing to their compact design, relatively low cost of fabrication, decreased sample volumes required for analysis, and high signal-to-noise ratio of single-molecule detection (72, 73). Applications of fluorescence imaging in microfluidics range from studies on the kinetics of proteins via single-molecule fluorescence resonance energy transfer (smFRET) (74) to observing the axonal transport of single nerve growth factors (75).

Fluorescence imaging is extremely well suited for probing single molecules in nanofluidic channels, which offer even smaller sample volumes to confine, manipulate, or immobilize individual molecules. For example, DNA has been elongated in a nanofluidic channel to probe its polymeric properties with fluorescence (76). Lim et al. (77) stretched DNA molecules to investigate individual nucleotides with fluorescent tags that selectively bind to methylated nucleotides, thereby distinguishing them from nonmethylated nucleotides. DNA molecules were also immobilized within a nanochannel to restrict their motion and observe DNA-repair proteins traveling along the DNA chain (78). Wu et al. (79) produced a microfluidic resistive-pulse sensing device with a single pore and collected simultaneous fluorescence and resistive-pulse signals. A percentage of the particles was not fluorescently labeled, for which only a resistive-pulse signal was detected. The combination of these two detection methods provided additional insight into fluorescence measurements that would otherwise go unnoticed.

**Figure 6:**
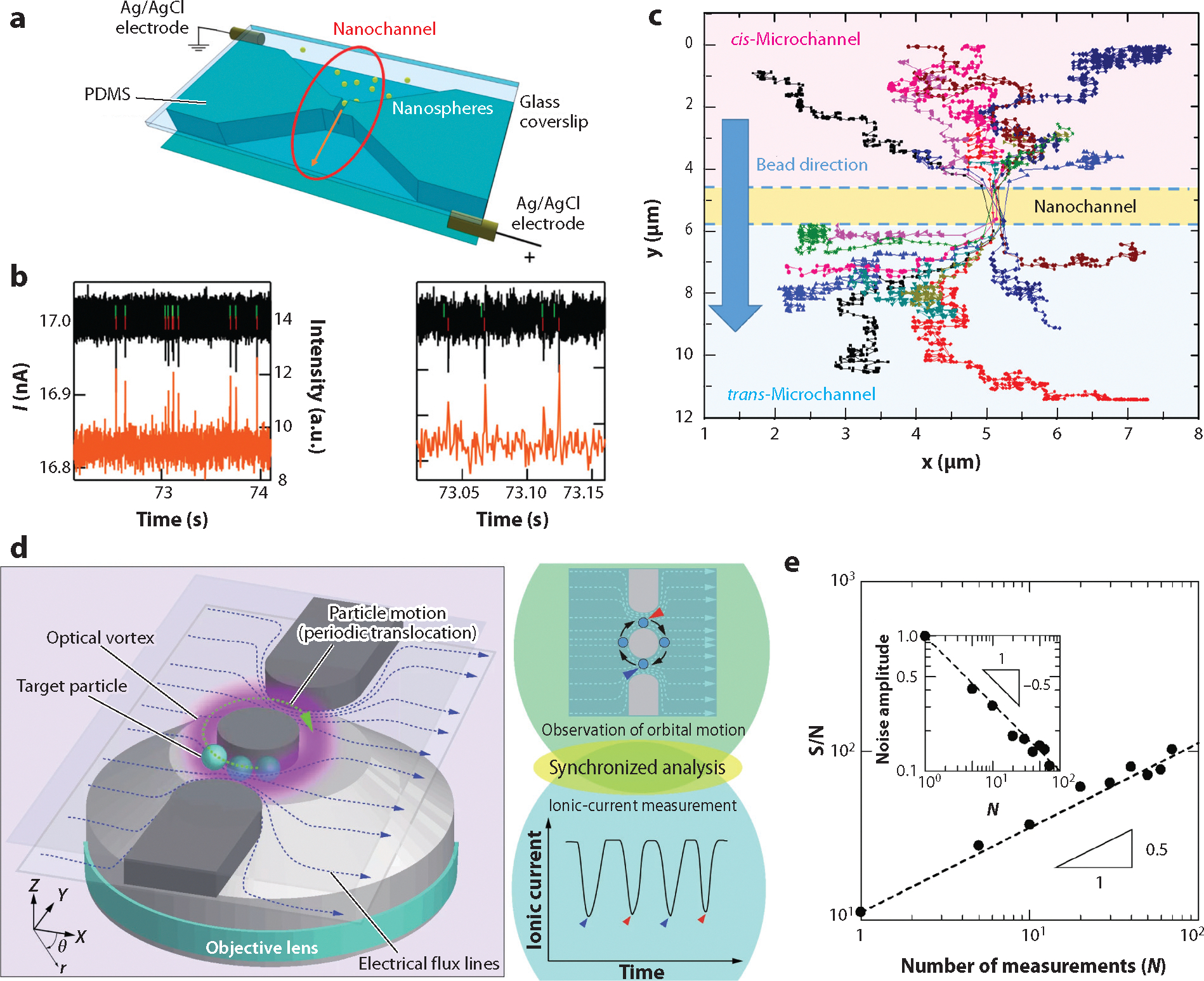
Resistive-pulse sensing coupled with optical techniques. (*a*) Schematic of a nanofluidic device for simultaneous measurement of current pulses and fluorescence as labeled nanoparticles translocate through a nanochannel (indicated by *orange arrow* in *red circle*). (*b*) Temporally correlated current pulses (*black*) and fluorescence bursts (*orange*) from nanoparticles translocating through the nanochannel in panel *a*. Each correlated event is indicated by a green dash (current) and red dash (fluorescence). The right panel is an enlarged section of the time window with the same *y*-axis scale. (*c*) Particle trajectories from tracking the fluorescence of nanoparticles. Each line of colored symbols represents the trajectory of an individual particle. Panels *a–c* adapted with permission from Reference 35; copyright 2015 American Chemical Society. (*d*) Schematic of a device with two nanopores located on opposite sides of a cylindrical pillar. Nanoparticles are trapped with an optical vortex and oscillate around the pillar to acquire multiple current pulses from a single particle. Each cycle around the cylindrical pillar generates two pulses per particle, indicated by the blue and red arrows. (*e*) Variation of the S/N with number of measurements (*N*). Panels *d*, *e* adapted with permission from Reference 81 (CC BY 4.0). Abbreviations: PDMS, poly(dimethylsiloxane); S/N, signal-to-noise ratio.

Angeli et al. (35) used an in-plane nanofluidic device to observe translocation of 40-nm fluorescently labeled nanoparticles simultaneously with resistive-pulse sensing and high-resolution fluorescence imaging, referred to as electro-optical nanoparticle tracking. The size and dwell time of the nanoparticles are determined by resistive-pulse sensing in the nanochannel (Figure 6a), and particle trajectories in the regions adjacent to the nanochannel are tracked with fluorescence imaging. The current pulses and fluorescence intensities from the nanoparticles entering, passing through, and exiting the nanochannel were highly correlated (Figure 6b). From the fluorescence imaging, the particle trajectories are precisely mapped (Figure 6c) and depict particle motion before capture and after release by the nanochannel. Diffusion coefficients of the particles inside and outside the nanochannel were estimated from the electrical measurements and fluorescence measurements, respectively, and results from the two methods agree well.

Particle position within a pore impacts both the pulse amplitude and dwell time of resistive-pulse measurements (80). Polystyrene beads (10 μm in diameter) were directly imaged as they traversed a pore that was 30 μm wide and 20 μm deep while the current was simultaneously recorded. Beads that traveled on-axis and remained close to the center of the pore produced pulse amplitudes that were approximately 90% of the pulse amplitude of beads that were close to the pore wall. This discrepancy in pulse amplitude is attributed to an increase in the distortion of the electric field near the walls, which leads to an increase in pulse amplitude. Moreover, the lateral position of the particle impacts the dwell time of a particle traversing the pore region. Beads positioned at the center of the pore travel ~3 times faster than beads close to the wall due to the pressure driven flow within the pore.

Simultaneous optical trapping and resistive-pulse measurements of particles were used to detect the same particle multiple times and collect additional information aside from particle size. As discussed above, averaging multiple measurements of a single particle improves the precision of the particle size measurement and signal-to-noise ratio. Nakajima et al. (81) trapped polystyrene beads ranging in diameter from 700 nm to 2 μm in an optical vortex and simultaneously conducted resistive-pulse measurements of the trapped particles (Figure 6d). The device had two pores: one pore positioned on each side of a cylindrical pillar. A trapped particle oscillated in a circular pattern around the pillar and passed through both pores during each cycle. The particle remained trapped until enough resistive pulses were collected. The signal-to-noise ratio increased with the number of measurements (Figure 6e).

Tsutsui et al. (82) constructed devices that contained a pore with a low aspect ratio (400 nm wide and 50 nm deep) to increase the ratio of access resistance to the pore resistance and to trap nanoscale particles with electric fields. As a particle approached the pore entrance, the access resistance increased, resulting in a decrease in measured current. Because the particle was too large to enter the pore, the particle became trapped at the pore entrance. The electrophoretic force that a particle experiences is proportional to the surface charge or ζ-potential of the particle. Therefore, a particle with a higher ζ-potential is trapped closer to the pore opening, which results in a larger decrease in the measured current.

## THE FUTURE OF IN-PLANE RESISTIVE-PULSE SENSING

In-plane resistive-pulse sensing will continue to evolve as researchers find ways to detect and examine smaller biomolecules and more complex samples, as well as integrate resistive-pulse sensing with other micro- or nanofluidic functional elements to simplify or improve upon existing measurements. Genomic and protein sequencing appear to be ideal fits for in-plane resistive-pulse sensing, which offers sensitivity, throughput, and integration with upstream and downstream processing. Challenges associated with making such precise measurements have been thoroughly discussed (83), although biological nanopores stably immobilized in a membrane have returned impressive results in both areas (84, 85). One addressable challenge is to reduce pore volumes of in-plane nanopores. High-resolution gas field ion sources (86) as a replacement for liquid metal sources have already created arrays of nanopores with sub-10-nm diameters and <10% variability in a membrane (87). Adaptation to in-plane designs would be minimal, and milling times could be optimized by selectively milling regions of critical components. Other strategies for reducing pore volumes may involve integration of biological nanopores within in-plane architectures or thinning or shrinking of preformed nanopores.

Integration of microfluidic components, especially separations, prior to resistive-pulse sensing looks particularly attractive for realizing multiplex analyses of complex mixtures. As previously stated, in-plane nanoscale architectures are highly adaptable to analyte size. However, complex mixtures with multiple analytes of interest, which vary significantly in size, present the possibility of clogging a device if the pore system is ill-suited for larger particles. With an upstream separation element, bands of analytes can be selectively routed to detection regions tailored to that target analyte.

**Figure 7:**
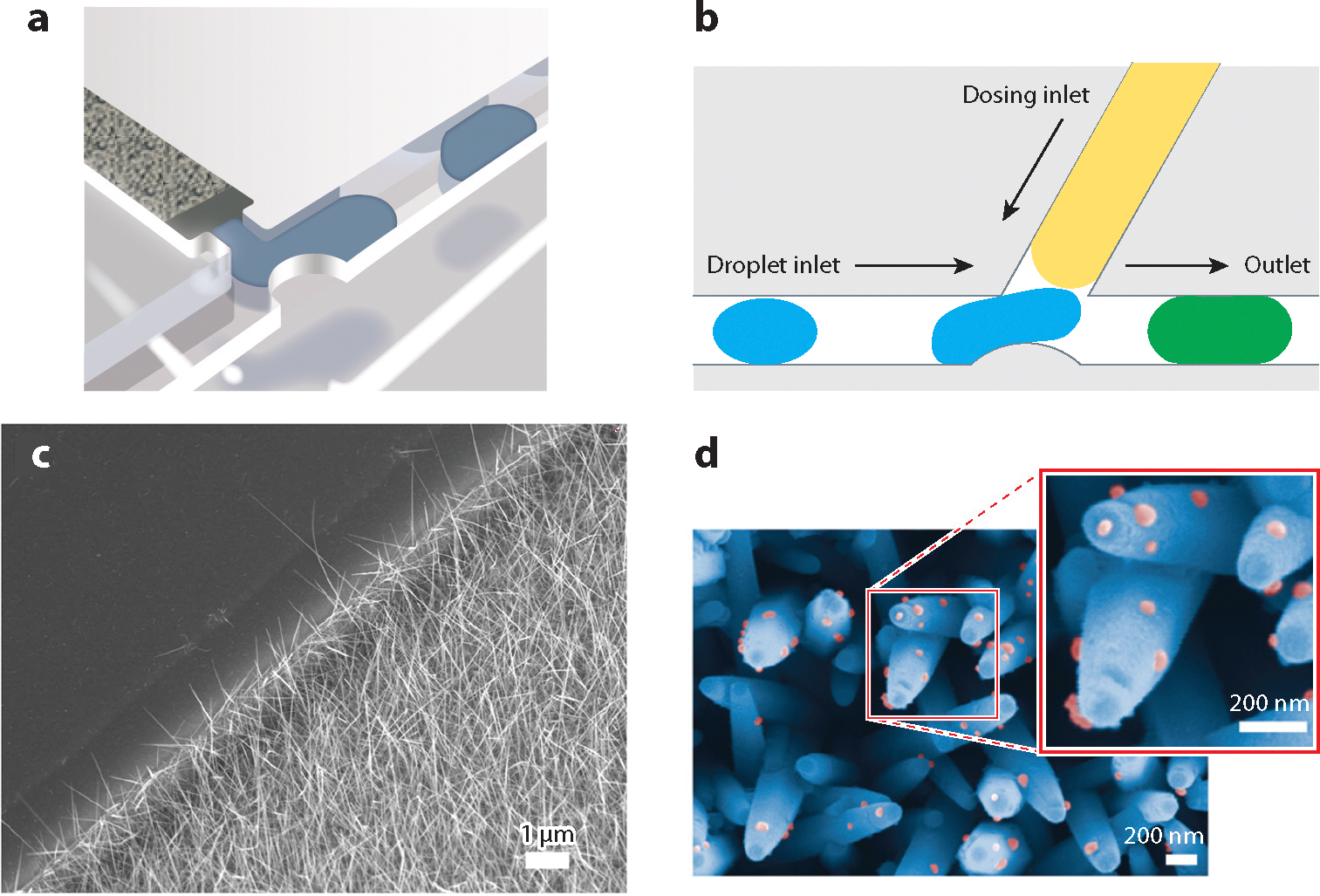
Microfluidic devices for the separation and isolation of samples. (*a*) Schematic of a microchannel packed with a C18 phase for liquid chromatography and coupled with droplet formation at the channel exit. (*b*) Microfluidic mixing tee to combine reagents with droplets formed in panel *a* for downstream processing. Blue droplets from the droplet inlet are mixed with a yellow fluid from the dosing inlet to produce green droplets at the outlet. Droplet manipulation demonstrates feasibility of a sample injection method for resistive-pulse measurements. Panels *a*, *b* adapted with permission from Reference 89; copyright 2017 American Chemical Society. (*c*) Scanning electron microscope (SEM) image of SnO_2_ nanowires grown in a microfluidic channel. Panel *c* adapted with permission from Reference 92 (CC BY 4.0). (*d*) SEM image of ZnO/Al_2_O_3_ core-shell nanowires (*blue*) chemically modified to capture extracellular vesicles (*pink*). Panel *d* adapted with permission from Reference 93 (CC BY 4.0).

Numerous analytical separation systems, particularly electrophoretic and chromatographic separations, have been transitioned to microfluidic devices. Some examples include an open-tubular reverse-phase chromatography system within a micro- and nanochannel network in which a complex mixture of 17 amino acids was separated (88); a microfluidic high-pressure chromatography system featuring a microchannel packed with C18 beads, which successfully separated coumarin-120 and three polyaromatic hydrocarbons (89); and a microfluidic capillary electrophoresis channel that separated various amino acid residues, including a d/l-glutamine chiral pair with a secondary in-line separation (90). The latter two examples incorporated droplet formation downstream of the separation for on-device fractionation, which compartmentalized the fractions and reduced deleterious effects from molecular diffusion (Figure 7a,b). Gerhardt et al. (89) even instituted a second droplet-microchannel interface, which added a fluorescence quenching agent, for downstream processing of specific fractions (Figure 7b). This downstream microchannel–droplet interface could be repurposed as a method for sample injection into a resistive-pulse sensor.

An isolation method that aligns well with in-plane resistive-pulse sensing is analyte-specific capture by chemically modified nanowires grown within microchannels (91, 92) (Figure 7c). Metal oxide nanowires have isolated both extracellular vesicles and cell-free DNA from liquid biopsy fluids (93, 94) (Figure 7d), and DNA is released following addition of EDTA to the solution (94). If distinct regions of nanowires, selective for a specific analyte, are placed within a microfluidic flow of a complex mixture (e.g., urine or blood) directly adjacent to a resistive-pulse sensing region, multiple analytes could be isolated from the mixture. Upon sequential release, these individual analytes could be detected with resistive-pulse sensing, tailored to each type of biomolecule, until the reserve of captured analyte is exhausted. Samples could be reloaded or recycled multiple times for improved throughput of the multiplexed analysis.

The combinations of micro- and nanofluidic elements are seemingly endless and leave the future of in-plane resistive-pulse sensing only limited by the imagination of the researcher. The possibilities noted above do not account for future advances in electronics and beam technologies that are sure to arise.

## SUMMARY POINTS

1. With the in-plane format, virtually any two-dimensional architecture of nanofluidic conduits can be conceived and fabricated.
2. The in-plane format seamlessly integrates resistive-pulse sensing with up- and downstream functional elements, including mixers, filters, reactors, separators, and sorters.
3. Coupling of resistive-pulse sensing with other functional elements in plane provides spatial precision on the nanometer scale and temporal precision on the microsecond to millisecond timescale.
4. Focused ion beam (FIB) milling fabricates nanofluidic channels and pores with nanometer precision and three-dimensional control.
5. With the in-plane format, resistive-pulse sensing can be easily coupled with optical techniques to measure biophysical properties of particles simultaneously.

## FUTURE ISSUES

1. As fabrication techniques continue to improve, smaller and more advanced features can be fabricated with greater precision.
2. More complex integrated devices will be designed and fabricated to tackle more advanced measurements at the nanoscale.
3. Reliable fabrication of nanofluidic devices in polymer substrates and development of alternative strategies to modify their surfaces will broaden the use and range of possible applications of nanofluidics.
4. In-plane nanofluidic devices will be coupled with other measurement techniques, such as electrochemistry and mass spectrometry.
